# Challenges Associated with Parenting While Incarcerated: A Review

**DOI:** 10.3390/ijerph18189927

**Published:** 2021-09-21

**Authors:** Monika Dargis, Arielle Mitchell-Somoza

**Affiliations:** 1 Department of Psychiatry, Weill Cornell Medical College, New York, NY 10065, USA; 2 Department of Psychology, Widener University, Chester, PA 19013, USA; akmitchell@widener.edu

**Keywords:** incarceration, parenting, prison

## Abstract

Of the 2.5 million people who are incarcerated in the United States, over half are parents. While it is well-established that incarceration has a detrimental impact on the children of incarcerated parents, less is known regarding the psychological impact of incarceration on parents themselves. The present review summarizes existing literature on the impact of incarceration on parents retrieved via online databases. Published articles were classified according to their overall themes and summarized. Pertinent studies include the psychological and emotional consequences of incarceration on parents, the experience of parenting while incarcerated, including barriers to parenting, the utility of parenting program interventions during periods of incarceration, and how these results differ for mothers and fathers. While the existing evidence introduces these issues, there is a need for additional research on the impact of incarceration on parents. These areas for future research as well as clinical implications are discussed.

## 1. Introduction

Over 2.5 million people are incarcerated in the United States, a rate of incarceration that far exceeds any other country in the world [1,2,3]. It is well documented that incarceration is associated with a wide range of adverse consequences, such as poor physical and mental health outcomes, difficulties obtaining employment, and socioeconomic hardship [4,5,6]. There is a growing recognition that incarceration also has significant negative effects on the family structure, a notable point given that over half of the individuals incarcerated in the US are parents [7,8]. Over the last several decades, researchers have started to parse apart how periods of incarceration influence the children and family members left behind. Perhaps not surprisingly, this work has demonstrated the long-term consequences that incarceration can have on the family unit. Scholars have also offered potential remedies to ameliorate consequences to children, such as parenting courses during periods of incarceration. However, comparatively less work has been performed on how incarceration affects the parents themselves who are incarcerated.

Separation from children, due to incarceration or other factors, represents a significant acute and long-term stressor [9]. There is evidence that such separation is linked to institutional misconduct (e.g., violating institutional rules) and psychiatric distress among incarcerated parents [10,11]. The extent to which separation distress contributes to such risk may be dependent on a number of other factors, such as availability of an alternative caregiver, support within the institution, and the nature of parent–child relationships prior to incarceration [12]. To the extent that navigating relationships with children is a significant stressor for parents who are incarcerated, this may represent a unique service need for this population. 

It is also important to explore gender role differences within the context of incarceration. Women tend to take on a larger role in terms of childcare duties prior to involvement with the criminal justice system and are more often primary caretakers of children than men. Therefore, mothers who are incarcerated may experience great stress associated with child separation [13,14]. Moreover, there is evidence that child and parent gender are both associated with differential outcomes for the child. For example, Burgess-Proctor and colleagues [15] reported that same gender parent–child dyads have the largest impact on children’s future involvement in the criminal justice system. These results suggest that the salience of parental incarceration is related to the gender of both the parent who is incarcerated and their child(ren). Accordingly, it may be the case that parent and child gender are important variables to consider when designing and implementing programming within correctional institutions.

Finally, while studies have shown benefits to implementing parenting programs in jails and prisons, recent work has shown that these programs may not be contextually relevant [16]. Although these programs may provide useful skills and information about how to effectively parent children once released from jail or prison, parents who are incarcerated are given little tangible information about how to effectively engage with their children from an institution and manage oftentimes complicated caregiver dynamics that may present as a result of incarceration.

Accordingly, the current article aims to summarize the current research on how incarceration affects parents themselves, emphasizing barriers to parenting while incarcerated, issues of mental health, gender role differences, parenting support programs and areas for future research.

## 2. Characteristics of Incarcerated Parents

Over two million individuals in the United States are incarcerated in jails and prisons, and more than half of this population has a history of mental health problems, including substance use [17]. Exposure to trauma is astronomically high among people who are incarcerated [18], particularly women who are incarcerated [19]. There is also a growing recognition that the systemic racism inherent in the United States criminal justice system is in and of itself a traumatic experience for people of color who are justice involved, who comprise a disproportionate percent of the system [20]. On average, an individual who is incarcerated within the US prison system has experienced extensive socioeconomic adversity, exposure to violence and trauma, mental health symptoms, struggles with substance use, racism and inequality, all by time a judge has allocated a criminal sentence. The additional stressor of being a parent forcibly separated from their children is yet another stressor placed on parents who are incarcerated.

It is important then to obtain a better understanding of who comprises this population. In state prisons, 48% of black males, 51% of Hispanic males, and 40% of white males report having a minor child, whereas 50% of black females, 62% Hispanic females, and 60% of white females report having a minor child. Considering federal prisons, 64% of black males, 64% of Hispanic males, and 34% of white males report having a minor child. Comparatively, two-thirds of Hispanic (67%) females in federal prison were mothers with minor children, compared to about one in two white (49%) and black (54%) females [8]. Children of color are significantly more likely to have a parent in prison than white children [8]. Parents incarcerated in state or federal prisons have, on average, two children each, resulting in over 1.5 million children with a parent currently in prison, and up to 5 million children who have ever had a parent incarcerated [21]. The children of parents who are incarcerated tend to be grade school-aged, with the average being 9 years old [22]. It is estimated the majority of parents lived with their children prior to their incarceration, although this is nearly twice as likely for mothers as compared to fathers [23].

Although similar by certain metrics, the United States jail population represents a unique subset of justice-involved people [24]. Generally speaking, the demographics of the jail population have shifted in recent years, with a sharp increase of women in jails, and a decrease in the racial disparities observed across the justice system. Additionally, there has been an increase in incarceration trends in more rural areas, whereas urban areas have shown some efforts toward decarceration [24]. Of parents who are incarcerated in jails, mothers report lower socioeconomic status and are more likely to identify as non-white when compared to a peer group of non-incarcerated mothers [25]. Similarly, studies have shown that under half of fathers incarcerated in jails have completed high school or an equivalent degree [26]. On average, parents in jails have more than one child, with most children under the age of 18 [26,27]. Up to 30% of incarcerated mothers report their children are under 5 years old [27], whereas fathers report an average child age of 7 [26]. Mothers in jails, compared to fathers, are more likely to have lived with their children as a primary caretaker prior to incarceration [28]. Additionally, due to the acute and often short-term nature of a jail stay, it is not uncommon for women to begin a period of incarceration while they are pregnant [25]. Compared to parents who have never been arrested, parents who have experienced a period of jail time report higher rates of substance use disorder and socioeconomic disadvantage [29,30].

## 3. Barriers to Parenting While Incarcerated

It is understood that parenting from behind bars is fraught with challenges [31]. Most concretely, institutional policies by definition limit the frequency and quality of contact between parents and their children. Accordingly, periods of incarceration significantly disrupt the frequency of contact between parents and their children. This is true for both jails and prisons, although each setting presents unique barriers to family contact. Jails are short-term holding facilities for people recently arrested, awaiting trial, or serving a sentence of typically less than 1 year. In contrast, prisons house individuals post-conviction who are serving sentences of greater than 1 year. By nature of these systematic differences, jails tend to have higher security requirements, and individuals housed in jails are often experiencing more acute psychiatric concerns (e.g., acutely psychotic, under the influence of a substance, experiencing withdrawal) when compared to individuals in prison [32]. Not surprisingly, these differences affect the extent to which parents who are incarcerated are able to communicate with their children. 

Individuals housed in jails can call and write to family members, and jails often allow for in-person visits. Although due to security concerns, visits may happen via Plexiglass barrier such that physical contact is not possible or are only available through video rather than live in-person [33]. Generally speaking, prisons offer more flexibility with in-person visits than jails (e.g., allowing physical touch) and also utilize video visits. However, prisons are often located far from metropolitan areas, which can affect the ability of family members to visit [32,34]. Proximity to family is not routinely a priority when determining the placement of an individual sentenced to prison [10]. Accordingly, once in prison, an individual who is incarcerated may be housed hours away from their nearest family, making in-person visitations burdensome or impossible, given limited financial and transportation options of the family [10].

Visiting practices and contact policies vary across jail and prison institutions widely, although typically allow for telephone calls, email, written correspondence, and in-person visits. The frequency and duration of phone calls and in-person visits are determined by the institution, and often entail a number of security requirements, which can negatively affect the overall connection [35]. For instance, phone calls and letters may be monitored by correctional staff, reducing privacy and comfort in communication between family members [36]. Similarly, in-person visits are monitored by correctional staff, and there may be limitations on the extent to which family members can physically touch each other. Although institutions may allow for phone calls and email correspondence, most require specific contact lists to be approved, with only a certain number of contacts allowed. This means that the individual who is incarcerated is limited in who they can speak with at any given time, and the institution may deny their contact list altogether [37]. This becomes particularly relevant for parents trying to stay in contact with their children, as there are any number of reasons why an institution will not approve a contact, which will then negatively affect the parent’s ability to communicate with their child [37]. 

For instance, a father who has recently perpetrated intimate partner violence may not be able to contact the victim of the violence, for understandable reasons. However, if the victim is also the primary caretaker of his children, this may mean he is essentially unable to communicate with the children [37]. While dynamics such as this are complex to navigate, this example highlights that the technical ability to make a phone call from prison does not mean parents are immediately able to interact with their children. Moreover, most institutions charge the person who is incarcerated and/or their families a fee for phone calls and email exchanges [38], which can present an additional financial barrier to parents maintaining contact with their children. A study conducted by Grinstead, Faigeles, Bancroft & Zack [39] estimated that women spend upward of $300 a month to maintain contact with their partners who are incarcerated, including in-person visits, phone calls, and gift packages. 

Relational challenges have also been highlighted as a factor that can affect the ability for parents and children to remain in contact during a period of incarceration. For instance, parental contact with children during incarceration is highly dependent on the caregiver of the child(ren) [40]. Outside of the functional considerations and burdens placed on the caregiver, the parent’s relationship with the caregiver is critical to facilitate contact with their child, particularly for younger children, who may not have the ability to receive phone calls on their own. Parents who have conflictual relationships with their children’s primary caregivers report experiencing additional challenges in staying connected with their children. For example, it has been documented that fathers who are incarcerated tend to report a lot of caregiver conflict, given that their children’s caregiver is typically the children’s mother, whereas mothers who are incarcerated tend to rely on other family members to care for their children during incarceration [41]. If the two parents are no longer in a romantic relationship and/or are experiencing relationship strain because of the incarceration, this can have a strong and negative impact on the father–child relationship [41]. Additionally, it has been reported that parents who are incarcerated and home caregivers may have different perspectives on the quality of the co-parenting alliance. For example, parents who are incarcerated reported more frequent letter writing and phone contact with their children in comparison to caregiver estimates [42]. More generally, if logistical barriers can be overcome, family dynamics can create additional challenges when parenting from prison [9]. 

Another barrier to parenting from prison that must be considered is the extent to which parent contact with their child is beneficial for both parties [43,44]. Although in-person visits may hold significant benefits for parents who are incarcerated and their children, in-person visits may also exacerbate existing parent–child relational issues. Klein, Bartholomew, and Hibbert [45], for instance, reported that parents who are incarcerated experienced high levels of disengagement and low cohesion among their family units prior to incarceration when compared to non-incarcerated parents. These pre-existing conflictual relationships are likely to be exacerbated by periods of incarceration. Moreover, visitation rules that inhibit family interactions, such as needing to be separated by glass, may be emotionally difficult for both the parent and their children because they are unable to physically connect [46]. Similarly, children and parents who are incarcerated may re-experience traumatic separation during visits, which may ultimately result in children and family members not wanting to visit [47]. There is also considerable stigma surrounding criminal justice involvement [48], which both the parents and their children may experience. While considerable literature on stigma and incarceration have focused on the negative consequences a criminal record has upon release (e.g., housing, employment [49]), stigma may also be experienced during incarceration, which may influence family contact. For instance, children may be bullied or teased by peers [48], which can affect their willingness to visit parents. Similarly, parents often feel considerable shame related to their incarceration and may choose not to have their children visit at all [50]. If high quality in-person visits are possible, (which in and of itself is subjective and difficult to measure), it is possible for parents who are incarcerated and their families to become upset or experience painful emotions.

Nonetheless, there is a plethora of evidence that visits by families during incarceration are beneficial for parents and their children. Specifically among mothers, Ref. [51] reported that a combination of generous visitation policies and proximity of the prison to the homes of home caregivers promoted mothers’ who are incarcerated feelings of attachment to their children. Consistent parent–child contact throughout periods of incarceration is a predictor for reunification upon release [52]. Additionally, there is evidence that family visits during incarceration reduce the likelihood of recidivism upon release [53], again highlighting the benefit of parents remaining connected with their children. Similarly, there is considerable evidence that children benefit from remaining in contact with their parents during a period of parental incarceration. For instance, McLeod and Bonsu [54] highlighted that remaining in contact with incarcerated fathers gave children a sense of stability and reduced trauma around the separation. Similarly, children who remain in contact with their incarcerated parent(s) have shown improvement in academic performance [55]. Additionally, children who have no contact with their parents often report feelings of isolation and alienation [43], whereas those who remain in contact show lower levels of anxiety [46].

Overall, it seems there are significant benefits for parents and their children to remain in contact throughout a period of incarceration, although there are also documented emotional consequences. It is likely that thoughtful discussions with the parents who are incarcerated and their families around the benefits and possible consequences of remaining in contact with children and how to best accomplish this is an important supportive service that institutions can provide parents. In other words, facilitating family visits and providing services to help families navigate the complexities of incarceration are important policy considerations for correctional facilities and community agencies [35].

## 4. Psychological Impact of Incarceration on Parenting

Given the lack of control and forced separation associated with incarceration, it is not surprising that many parents who are incarcerated report experiencing psychological distress in addition to aforementioned mental health concerns prevalent in this population [56]. Parents who are jailed and have young children report elevated rates of depression and thought problems, such as hallucinations, strange thoughts, and self-harming behaviors [56]. Among jailed parents, rates of mental health symptoms are three to five times that of the general population, including high rates of comorbidities [56]. Although those who are incarcerated experience higher rates of mental illness than the general population on average, it appears that parents who are incarcerated may face additional and unique psychological distress [57]. Stressors associated with parenting may include general worry about their children, lack of control associated with forced separation, caregiver conflict, custody issues, concerns regarding transparency about their criminal behavior, and concerns surrounding loss of identity as a parent [11,58]. These types of parenting stressors have been associated with more depression and anxiety symptoms, more frequent institutional misconduct, and more self-reported in-prison aggression for parents who are incarcerated [10,11]. A sense of relationship disconnection and infrequent contact with children are particular drivers for depressive symptoms, particularly among mothers who are incarcerated [58].

An additional and specific stressor that affects the wellbeing of parents who are incarcerated is self-perceived competence as a parent, which has been linked to elevated levels of anxiety and depression [11,59]. Parents who report lower levels of competence in their parenting abilities also tend to have more difficulties with institutional adjustment, including higher rates of misconduct [10]. Accordingly, acute psychological distress associated with separation from their children is an important consideration for institutions, for both mental health and security reasons.

Scholars have theorized that disruptions in attachment during a period of parental incarceration may make this separation a particular risk factor for the development of psychopathology in children [60,61]. The model of attachment, originally proposed by Bowlby [62], highlights the importance of children feeling secure and safe with their caregivers, for them to feel safe navigating their environments. Without this secure base, Bowlby posited that children are at risk of adverse outcomes, and research has demonstrated that individuals who have insecure attachments to their caregivers are more likely to experience psychopathology later in life [63].

Although discussion around attachment issues related to parental incarceration has understandably focused on the attachment consequences for the children, it is a relevant model for the parents who are incarcerated as well. First, adults who are incarcerated have often experienced incarceration of their caregivers as children [64], suggesting that any adverse attachment consequences experienced in childhood may be exacerbated when separated from their own children. Second, it is well documented that rates of trauma exposure and adversity are high among people who are justice involved, which also has implications for attachment insecurity [18,65]. Studies have shown that individuals who are incarcerated generally report insecure or otherwise disorganized attachment styles [66,67]. It is plausible then that the unique distress parents who are incarcerated experience related to separation from their children stems in part from long-standing insecure attachment patterns.

Parental stress experienced by parents who are incarcerated not only uniquely impacts overall psychological wellbeing but may also impact overall relational wellbeing and institutional conduct. The existing literature further suggests differential stressors (e.g., relational, emotional, and social) among parents who are incarcerated that may result in unique psychological needs during periods of incarceration.

## 5. Gender Differences between Incarcerated Mothers and Fathers

Regardless of criminal justice involvement, women tend to take on greater childcare responsibilities within relationships, and mothers typically act as the primary caregivers of children [13,14]. For instance, among parents in state prison who had lived with their minor children just prior to incarceration, mothers (77%) were almost three times more likely than fathers (26%) to report that they had provided most of the daily care for their children [8]. Not surprisingly, this gendered dynamic has significant implications when considering the effect of incarceration on mothers versus fathers. This is in part because women experience a higher rate of mental health and substance use disorders than men, overall [68], which is especially true of justice-involved women [69]. It has been noted that pre-existing stressors, such as mental health diagnoses, often have a larger impact on mothers who are incarcerated than fathers who are incarcerated [8]. Moreover, many mothers report that the forced separation from their children during a period of incarceration is not only stressful but is experienced as traumatic. Given the overwhelmingly high rates of trauma that women who are justice involved experience throughout their lives [70], the consequences of being separated from their children may exacerbate and/or reinforce pre-existing trauma-related symptoms. It has been documented that mothers experience an intense distress during the initial period of incarceration [71], although the acuity of the distress tends to dissipate with time. This would suggest that mental health screenings and intake procedures should take into consideration women’s motherhood status and take time to assess how separation from children may be exacerbating emotional distress [44]. However, although women’s motherhood status may impact levels of emotional distress throughout periods of incarceration, mothers may also be hesitant to share this information with correctional staff due to various concerns (e.g., child protection involvement, children’s or their own safety, etc.)

While the initial period of incarceration may represent the most apparent distress for mothers, it is also known that parental stress and conflicts with their children’s caregivers contribute to depression symptoms throughout an incarceration [60]. Notably, women who did not live with their children prior to their incarceration report considerable stress in being unable to fulfill a parental role in their children’s lives while incarcerated [56]. Although men also report significant stress associated with parenting [10], it has been documented that certain men view incarceration as a “dormant” period for fatherhood, with hopes of a “fresh start” when they re-enter the community [71]. In contrast, mothers have more frequent and sustained contact with both their children and their children’s caregivers throughout their incarceration when compared to fathers who are incarcerated [10], suggesting that women continue to have a greater parenting responsibility from prison than men.

Research has consistently demonstrated the adverse psychological impact of incarceration on fathers as well. Active fatherhood (e.g., establishing paternity, providing financial support, and participating in physical and emotional care) is challenging to enact during periods of incarceration [72]. Fathers who are incarcerated often have less contact with children compared to mothers who are incarcerated [10] and receive less social support [73]. Experiences of incarceration may have a strong impact on some fathers’ self-concept. For example, existing literature suggests that most men who are incarcerated have reported having lost their fatherhood identity [74]. Similarly, other studies have described men who are incarcerated as having repressed their fatherhood identity during imprisonment to cope with separation from their children [71,75]. Direct interviews conducted with fathers who are incarcerated noted that the fathers viewed their incarceration as a form of child neglect or abandonment [71]. Moreover, fathers who are incarcerated have been found to focus on their inability to perform fathering duties such as protection, support, guidance, and discipline. Outside of specific parenting duties and identity, it has been documented that fathers who are incarcerated express feelings of guilt and concern about the distress they have caused in their children’s lives. Similarly, many fathers who are incarcerated describe a sense of loss that they are not able to participate in their children’s lives [76]. Risk of depressive symptoms is of particular concern for men who feel detached from their children [77].

Considered together, these results indicate that stigma, emotional pain, and institutional barriers (e.g., phone access and visitation rules) affect incarcerated mothers and fathers similarly, although the consequences may differ. Mothers who are incarcerated face considerable parental stress and challenge given the high likelihood that they were a primary caretaker to their children prior to incarceration, and therefore specific gender-appropriate parenting accommodations are critical. However, it is also apparent that fathers who are incarcerated also experience considerable parental stress and are not unaffected by child separation. We may overlook the extent to which fathers play a caregiver role in their children’s lives at times, which may adversely affect the children when a father is removed from the home [78].

## 6. Programs for Incarcerated Parents

Programming available for individuals who are incarcerated, regardless of parental status, varies considerably across institutions, based on issues such as financial resources, priorities in funding, availability of staff, etc. Most institutions offer, and at times require, participation in corrections-based programming targeting behavioral changes associated with the individual’s criminal offense (e.g., sex offender treatment, anger management, domestic violence treatment). Additionally, individual- and group-based mental health services are routinely offered in institutions [79], although the availability and utilization of these services may be limited due to a shortage of mental health professionals and/or stigma associated with seeking mental healthcare [80].

Outside of these core programming components, correctional institutions across the world have also developed and implemented various parenting programs to help support parents who are incarcerated, and in certain cases, facilitate contact with their children. Parenting interventions that have been implemented in prisons across the United States typically involve weekly group sessions that teach parents about child development, stress management, and communication skills [81,82]. The most frequent program goal across parenting programs tends to be improving child–parent relationships by increasing general parent knowledge and skills, and the most recurrent topics covered in the classes tend to focus on discipline and general positive parenting concepts [16]. Programs specifically geared toward parents are often skills-based, and although certain programs may discuss topics such as mental health and substance use, they are not mental health or substance use programs. A review of prison-based parenting programs conducted by Purvis [83] found that, for children, parenting programs were associated with improved self-esteem, mental health, wellbeing, and academic performance and decreased truancy and delinquency. For parents who are incarcerated, parenting programs were associated with increased bonding with and empathy toward their child, enhanced knowledge of child development, and enhanced behavior management. Similarly, Wilson, Gonzalez, Romero, Henry, and Cerbana [84] reviewed the effectiveness of an education program for parents who are incarcerated, citing the numerous benefits of educational initiatives for parents themselves and their children. Specifically, it was found that both mothers and fathers who were incarcerated reported increases in self-esteem, parental knowledge, and positive attitudes toward parenting after participating in a parenting education program. Importantly, parenting programs may support parent–child relationships throughout periods of incarceration and may increase the likelihood of parent–child reunification upon release.

Although parent educational initiatives may seem promising, there are a number of caveats that should be considered. First, it has been reported that there is little consistency in program development and evaluations [85], making it difficult to develop evidence-based or empirically supported programs. Along these lines, considerable research to date has relied on self-report outcomes, which may attribute greater causality to the parenting program and positive outcomes than is occurring. Additionally, considerable research on parenting programming focuses on whether the program goals are achieved rather than whether the program curriculum is relevant to the carceral setting or perceived as positive and useful by participating parents. Accordingly, many of these parenting programs are not contextually relevant, are not created by individuals who have experienced incarceration, and do not address the unique needs of parents who are incarcerated [16,86]. 

An additional limitation to our understanding of the efficacy of parenting programs is the somewhat limited demographic representation. Most parenting programs have been created for and tested on predominantly European American middle-class samples [87]. Therefore, parenting programs that are considered “evidence-based” and implemented in diverse settings often have difficulty with recruitment and experience high levels of attrition due to the mismatch between the curriculum and cultural norms and contextual needs [88,89]. Parenting programs that address cultural, social, or historical factors that influence the experiences of the target population are found to have the highest effect size in improved parenting behavior [89]. To achieve the greatest outcomes for parents, children, and society as a whole, it is important that programs take the initiative to make their curriculum not only culturally but also contextually relevant to those participating [16].

Although limitations exist, there is available data regarding what aspects of parenting programs group participants find helpful. Previous studies have shown that specific topics such as coping with addiction, managing complex emotions, discipline, empathy, and positive parenting practices were rated as meaningful by parents in prison [90,91,92]. Additionally, program participants report opportunities to engage in practical activities as especially meaningful [93]. Moreover, previous research has described the relationship between program facilitators and recipients as having a significant impact on overall impact and engagement [94,95]. Promoting strong relationships between facilitators and participants may be of particular importance in a prison context, where distrust of professionals, unequal power dynamics, and a reluctance to engage in programs are common problems [96]. Without these considerations, participants may have a difficult time identifying with parenting programs, which may lead to frustration, dissatisfaction, and lack of engagement [97].

In addition to prison-based parenting programming, there are also studies that have examined programs and supports created for families upon community-re-entry. This is a particularly important consideration, given that many of the difficulties parents faced prior to their incarceration (e.g., socioeconomic disadvantage, lack of childcare, difficulty obtaining employment) remain challenges upon re-entry and can therefore negatively affect their ability to re-engage with their children [98]. It has consistently been recommended that mental health programs be available for recently released people to address pre-existing mental health concerns as well as provide support around the various stressors associated with community re-entry [58]. This is an especially strong recommendation for mothers who are re-entering, who often experience considerable emotional distress upon release about their perceived inability to parent in the context of many challenges [99]. It has been recommended that parenting programs, specifically for parents who are re-entering, not focus on parenting skills per se but focus on ameliorating the many barriers’ parents face when reuniting with their children (e.g., financial, mental health, coping skills, etc.) [100].

In part, re-entry support can come from pre-release services provided by the institution. Staff work with the incarcerated individual to identify various aspects integral to their re-entry, such as where they will live, what extended supervision requirements they might have, and facilitate conversations with family members to ensure everyone has the same expectations [101]. Similar to most other prison-based services, the quality and intensity of pre-release services vary across institutions; thus, while in theory, re-entry staff can be a major support for child re-unification, this may not always be feasible. Once officially released, certain jurisdictions offer specific services for parents. For instance, Washington State offers transitional living houses that allow children, thus enabling mothers to more immediately reunited with their children and actively engage as a parent [102]. Programming in Texas provides individual and family counseling sessions as well as support groups for individuals who are incarcerated upon re-entry [103], and programs throughout New York offer 24 h crisis intervention services and connects probation officers, case managers, and families together to effectively plan for re-entry [103]. Although a number of re-entry programs exist, many of which that cater specifically to parents, researchers have highlighted the lack of rigorous research available on the efficacy of these programs [104]. 

While there is certainly more work to undertake in fine-tuning evidence-based, culturally sensitive, and contextually relevant programming, the available evidence suggests that these programs are generally well received and can improve parents’ self-perceptions and attitudes toward parenting. To the extent that these attitude changes lead to meaningful behavioral change is less well defined, although this is likely an area of research that will need to be expanded as prison-based programs incorporate more context-relevant, readily applicable material.

## 7. Discussion

Incarceration policies in the United States have resulted in 45% of Americans with an immediate family member who is incarcerated [105]. This translates to over 2.5 million incarcerated people, of which over half are parents [8]. Accordingly, the aim of the current review was to summarize the extant literature on how incarceration affects the parents themselves. We have highlighted the many barriers parents face in remaining present in their children’s lives once incarcerated, the negative psychological consequences associated with parents being separated from their children, the nuanced and often gendered differences in how incarceration affects mothers and fathers, and the parenting programs many institutions have implemented in response to these needs. Below, several areas for future research are suggested.

Incarceration has immense consequences for family structures. Systems-level barriers (e.g., the institution itself) and interpersonal barriers (e.g., the relationship with the caregiver) affect the extent to which parents can have contact with their children. While the literature suggests that increased contact with children is largely beneficial for parents, both in terms of mental health and institutional adjustment [106], this must be balanced by mixed literature on how visiting parents in prison affects children [46,107]. More work must be undertaken to aid parents, caregivers, and institutions in their decision making around parental involvement during a period of incarceration. Although it may be the case that parents benefit from increased contact with their children, families must consider the nuance of balancing what is best for the child and the parent simultaneously.

Disconnection from children is a risk factor for adjustment disorder or other mental health symptom exacerbation during a period of incarceration. This seems to be particularly acute during the initial separation [60], suggesting that both jails and prisons should be attuned to parental status as a potential risk factor for psychiatric distress. Provision of mental health services throughout a period of incarceration for parents may be helpful, although it may be especially beneficial to ensure parental status is inquired about during the initial mental health screening and intake procedures within an institution to ensure adequate support and monitoring is provided. This may be an especially important intake consideration for mothers who are incarcerated, given that mothers are more often the primary caregivers of children and women in the criminal justice system are at higher risk of mental illness [69]. Although important to consider parental status, parents may be hesitant to disclose this status to correctional staff for any number of reasons, such as shame or guilt or fear of negative consequences.

The realities of providing specific and sensitive mental health services to parents who are newly incarcerated is likely complicated, however. Correctional facilities have an immense demand for mental healthcare and often face shortages of treatment providers [108]. Incorporating an assessment of impact on parents during the standard intake procedure will at least ensure awareness of acute distress, although ability to follow up with appropriate services may be somewhat limited depending on the institution. This is a critical issue facing the criminal justice system as a whole, regardless of parental status [108].

There is a growing literature citing the effectiveness of peer-led interventions and support groups among people who are justice involved [109]. For example, Ray et al. [110] documented self-reported improvements in mental and physical health following participation in a community-based, peer-lead re-entry program for individuals with substance use disorders. Similarly, it has been documented that peer-lead emotional support groups in prisons can help reduce the incidence rate of suicide among people who are incarcerated. Given that peer-lead groups seem to improve overall mental health among participants, this may be a service that could be leveraged to help parents who are incarcerated, specifically. Although further research would be required to demonstrate effectiveness, it may be that a peer-led support group for parents can ameliorate the initial distress associated with separation and reduce demand for mental health services. Moreover, parents may find that receiving practical advice and learning prison-specific parenting skills from people who are in the same situation may reduce the overall feedback that many parenting programs receive regarding their lack of applicability and relevance to the prison setting.

Outside of psychiatric symptoms that may develop among parents during a period of incarceration, it may be helpful to consider that many parents who are incarcerated experienced parental incarceration as a child. Having a parent incarcerated represents a risk factor for future incarceration among children [111]. Consequently, the individual who is incarcerated may have a complex reaction to their own confinement, including feelings of shame and/or traumatic childhood memories. Given the link between parental stress and institutional misconduct [11], as well as experiences of shame and criminal recidivism [112], it may be helpful to examine potential mediating links between parental stress, shame associated with perceived failure of parental duties, and mental and behavioral problems. This line of work can help guide programming designed for incarcerated parents.

Along these lines, considering parental incarceration through the lens of attachment theory may provide unique insights into effective treatments for both parents who are incarcerated as well as their families. Makariev and Shaver [61], for instance, outlined a model of attachment as it pertains to parental incarceration, including a number of intervention options specifically targeting attachment concerns. Although most of the interventions focus on reducing consequences of parental incarceration for children, there is evidence that these programs are associated with improved behavioral outcomes for the parents who are incarcerated as well [113]. It may be helpful for future research to examine attachment-focused programming for incarcerated parents that takes into consideration their own attachment needs and their current separation from children. One potential candidate may be emotion focused therapy (EFT) [114], an evidence-based treatment for individuals, couples and families that is rooted in attachment theory. Because EFT can be implemented for individuals or families, it may be a promising intervention for parents during or after incarceration, as well as their families. 

Along these lines, evidence-based, trauma-focused treatments for parents who are incarcerated may also be a viable intervention for the psychological distress associated with separation from children. Not only is the separation itself potentially traumatic, the high prevalence rate of trauma among people who are justice involved warrants specific intervention. Receiving treatment for trauma is particularly relevant for parents who are incarcerated, given that the behavioral consequences associated with trauma-related disorders such as posttraumatic stress (e.g., increased reckless behavior, irritability, social avoidance) may themselves be barriers to effective parenting during and after incarceration. 

Although mothers and fathers separation from their children may be nuanced, there is evidence that parents, regardless of gender identity, experience a level of distress due to separation from their children during incarceration, and that perceived incompetence in parenting is a key factor that exacerbates psychological adjustment [115]. Continuing to promote parenting programs across institutions, then, is important, especially given evidence that parenting programs seem to have positive effects on parents, such as increased confidence in parenting. However, individuals who are incarcerated often seek practical parenting advice that can be implemented from behind bars, rather than focusing solely on hypothetical strategies to be used at a certain point in the future when they are released. This is particularly true for individuals with lengthy sentences. Accordingly, it has been argued that for parenting programs to be efficacious, participants must have contact with their children during incarceration to give them an opportunity to practice learned skills and receive feedback in a supportive environment [16]. 

In addition to considering parental stress associated with incarceration, it is important to gain a better understanding of how a parent–child relationship progresses once the parent has been released, and how this impacts the parent. It is well known that the period immediately following release from prison is the highest risk time for those re-entering, both in terms of health and mental health risks as well as recidivism [116]. The stress people re-entering the community face is immense and can only be multiplied if they are returning to a caregiver role. There is evidence that men who were formerly incarcerated continue to struggle with their identity as a father following their release [117], and that women who are incarcerated struggle to stay involved in their children’s lives even with support during incarceration [51]. Outside of the stress associated with acclimating post-release, there continue to be many obstacles to parenting once released from prison or jail. Transitional housing and/or extended supervision requirements may limit the extent to which they have contact with their families. This may be more difficult for children to understand, and present unique challenges for the parent to navigate. It is important to gain more knowledge on how re-entry plans and probation/parole officers influence parenting relationships upon release.

Finally, we will be remiss without commenting on how an already difficult landscape of parenting in prison has been exponentially more difficult during the COVID-19 pandemic. Correctional institutions quickly became hot spots of COVID-19 infection, given close quarters and lack of personal protective equipment. As a result, federal and state-level institutions restricted access and movement, meaning that family members could no longer visit [118]. As we pass a year since the start of the pandemic, many institutions still have restrictions on visiting procedures. Certain facilities have responded to visitation restrictions by increasing access to phone calls (e.g., reducing the cost of calls) and starting to utilize video chat platforms to facilitate family connection. Although access to video chat platforms may present a unique set of security and privacy challenges, the ability for institutions to adapt (and the broader world to adapt to telehealth and remote working options) suggests that increasing use of technology in correctional institutions may be key to reducing barriers to familial contact, as well as access to mental health treatment. As COVID-19 restrictions ease, continuing the use of video chat platforms can facilitate more connected family visits, especially for those who live a significant distance away from the institution their family member is incarcerated in [119]. Moreover, implementing this type of technology can increase access to mental health care by utilizing more community-based resources via telehealth services. It will be important for researchers and clinicians alike to develop standards of best-practice use of video-based technology within institutions [120].

Although more research needs to be performed to better understand the nuances of how parents are affected by separation from their children, it is clear that parents who are incarcerated experience acute distress and long-term stress associated with parenting from prison. Continuing to provide relevant and evidence-based programming, as well as considering the mental health implications of family separation during incarceration, is critical to minimize consequences to both the children and the parents themselves.

## Data Availability

Not applicable.

## References

[1] Shlafer R., Duwe G., Hindt L. (2019). Parents in prison and their minor children: Comparisons between state and national estimates. Prison J..

[2] Nutt L.M., Deaton D., Hutchinson T. (2008). Children and Families of Incarcerated Parents: A Demographic Status Report and Survey.

[3] US Census Bureau (2005). US Census Bureau Annual Estimates of Population for US and States, and for Puerto Rico: 1 April 2000–1 July 2005 (NST-EST2005-01).

[4] Apel R., Ramakers A. (2018). Impact of Incarceration on Employment Prospects. Handbook on the Consequences of Sentencing and Punishment Decisions.

[5] Massoglia M. (2008). Incarceration, health, and racial disparities in health. Law Soc. Rev..

[6] Wildeman C., Wang E.A. (2017). Mass incarceration, public health, and widening inequality in the USA. Lancet.

[7] Aaron L., Dallaire D.H. (2010). Parental incarceration and multiple risk experiences: Effects on family dynamics and children’s delinquency. J. Youth Adolesc..

[8] Maruschak L.M., Bronson M., Apler M. (2021). Parents in Prison and Their Minor Children: Survey of Prison Inmate, 2016 (NCJ 252645).

[9] Beckmeyer J.J., Arditti J.A. (2014). Implications of in-person visits for incarcerated parents’ family relationships and parenting experience. J. Offender Rehabil..

[10] Loper A.B., Carlson L.W., Levitt L., Scheffel K. (2009). Parenting stress, alliance, child contact, and adjustment of imprisoned mothers and fathers. J. Offender Rehabil..

[11] Houck K.D., Loper A.B. (2002). The relationship of parenting stress to adjustment among mothers in prison. Am. J. Orthopsychiatry.

[12] Arditti J.A. (2016). A family stress-proximal process model for understanding the effects of parental incarceration on children and their families. Couple Fam. Psychol. Res. Pract..

[13] McBride B.A., Schoppe S.J., Rane T.R. (2002). Child characteristics, parenting stress, and parental involvement: Fathers versus mothers. J. Marriage Fam..

[14] Pedersen D.E. (2012). The good mother, the good father, and the good parent: Gendered definitions of parenting. J. Fem. Fam. Ther..

[15] Burgess-Proctor A., Huebner B.M., Durso J.M. (2016). Comparing the effects of maternal and paternal incarceration on adult daughters’ and sons’ criminal justice system involvement: A gendered pathways analysis. Crim. Justice Behav..

[16] Henson A. (2020). Meet them where they are: The Importance of contextual relevance in prison-based parenting programs. Prison J..

[17] James D.J., Glaze L.E. (2006). Mental Health Problems of Prison and Jail Inmates.

[18] Briere J., Agee E., Dietrich A. (2016). Cumulative trauma and current posttraumatic stress disorder status in general population and inmate samples. Psychol. Trauma: Theory Res. Pract. Policy.

[19] Komarovskaya I.A., Booker Loper A., Warren J., Jackson S. (2011). Exploring gender differences in trauma exposure and the emergence of symptoms of PTSD among incarcerated men and women. J. Forensic Psychiatry Psychol..

[20] Sawyer W. (2020). Visualizing the racial disparities in mass incarceration. Prison. Policy Initiat..

[21] Murphey D., Cooper P.M. (2015). Parents Behind Bars: What Happens to Their Children?.

[22] Bronson J., Maruschak L. (2016). An Overview of Prison and Jail Inmate Physical and Mental Health: National Findings from the Bureau of Justice Statistics. APHA 2016 Annual Meeting & Expo (29 October–2 November 2016).

[23] Johnson-Peterkin Y. (2003). Information Packet: Children of Incarcerated Parents.

[24] Jones A. (2020). Stagnant population and changing demographics: What the new BJS report tells us about correctional populations. Prison. Policy Initiat..

[25] Bell J.F., Zimmerman F.J., Cawthon M.L., Huebner C.E., Ward D.H., Schroeder C.A. (2004). Jail incarceration and birth outcomes. J. Urban Health.

[26] Shlafer R.J., Davis L., Hindt L., Weymouth L., Cuthrell H., Burnson C., Poehlmann-Tynan J. (2020). Fathers in jail and their minor children: Paternal characteristics and associations with father-child contact. J. Child. Fam. Stud..

[27] Kelly P.J., Peralez-Dieckmann E., Cheng A.L., Collins C. (2010). Profile of women in a county jail. J. Psychosoc. Nurs. Ment. Health Serv..

[28] Jensen V., DuDeck-Biondo J. (2005). Mothers in jail: Gender, social control, and the construction of parenthood behind bars. Sociol. Crime Law Deviance.

[29] Yi Y., Turney K., Wildeman C. (2017). Mental health among jail and prison inmates. Am. J. Men’s Health.

[30] Phillips S.D., Dettlaff A.J. (2009). More than parents in prison: The broader overlap between the criminal justice and child welfare systems. J. Public Child Welf..

[31] Massoglia M., Warner C. (2011). The consequences of incarceration: Challenges for scientifically informed and policy-relevant research. Criminol. Pub. Pol’y.

[32] Cramer L., Goff M., Peterson B., Sandstrom H. (2017). Parent-Child Visiting Practices in Prisons and Jails.

[33] Arditti J.A. (2012). Parental Incarceration and the Family: Psychological and Social Effects of Imprisonment on Children, Parents, and Caregivers.

[34] Rubenstein B.Y., Toman E.L., Cochran J.C. (2019). Socioeconomic barriers to child contact with incarcerated parents. Justice Q..

[35] Poehlmann-Tynan J., Pritzl K. (2019). Parent–child visits when parents are incarcerated in prison or jail. Handbook on Children with Incarcerated Parents.

[36] Arditti J.A. (2003). Locked doors and glass walls: Family visiting at a local jail. J. Loss Trauma.

[37] Weill J.M. (2016). Incarceration and Social Networks: Understanding the Relationships That Support Reentry.

[38] Severin M. (2003). Is there a winning argument against excessive rates for collect calls from prisoners?. Cardozo L. Rev..

[39] Grinstead O., Faigeles B., Bancroft C., Zack B. (2001). The financial cost of maintaining relationships with incarcerated African American men: A Survey of Women Prison Visitors. J. Afr. Am. Men.

[40] Norman J.A. (2014). Children of Prisoners in Foster Care. Children of Incarcerated Parents.

[41] Magaletta P.R., Herbst D.P. (2001). Fathering from prison: Common struggles and successful solutions. Psychother. Theory Res. Pract. Train..

[42] Loper A.B., Phillips V., Nichols E.B., Dallaire D.H. (2014). Characteristics and effects of the co-parenting alliance between incarcerated parents and child caregivers. J. Child. Fam. Stud..

[43] Shlafer R.J., Poehlmann J. (2010). Attachment and caregiving relationships in families affected by parental incarceration. Attach. Hum. Dev..

[44] Shlafer R.J., Loper A.B., Schillmoeller L. (2015). Introduction and literature review: Is parent–child contact during parental incarceration beneficial?. Child. Contact Incarcer. Parents.

[45] Klein S.R., Bartholomew G.S., Hibbert J. (2002). Inmate family functioning. Int. J. Offender Ther. Comp. Criminol..

[46] Dallaire D.H., Wilson L.C., Ciccone A. (2012). The family drawings of at-risk children: Concurrent relations with contact with incarcerated parents, caregiver behavior, and stress. Attach Hum. Dev..

[47] Nesmith A., Ruhland E. (2008). Children of incarcerated parents: Challenges and resiliency, in their own words. Child. Youth Serv. Rev..

[48] Murray J., Murray L. (2010). Parental incarceration, attachment and child psychopathology. Attach. Hum. Dev..

[49] Decker S.H., Ortiz N., Spohn C., Hedberg E. (2015). Criminal stigma, race, and ethnicity: The consequences of imprisonment for employment. J. Crim. Justice.

[50] Charles P., Muentner L., Kjellstrand J. (2019). Parenting and incarceration: Perspectives on father-child involvement during reentry from prison. Soc. Serv. Rev..

[51] Martin M. (1997). Connected mothers: A follow-up study of incarcerated women and their children. Women Crim. Justice.

[52] Tuerk E.H., Loper A.B. (2006). Contact between incarcerated mothers and their children: Assessing parenting stress. J. Offender Rehabil..

[53] Casey W.M., Copp J.E., Bales W.D. (2021). Releases from a local jail: The impact of visitation on recidivism. Crim. Justice Policy Rev..

[54] McLeod B.A., Bonsu J. (2018). The benefits and challenges of visitation practices in correctional settings: Will video visitation assist incarcerated fathers and their children?. Child. Youth Serv. Rev..

[55] Trice A.D., Brewster J. (2004). The effects of maternal incarceration on adolescent children. J. Police Crim. Psychol..

[56] Milavetz Z., Pritzl K., Muentner L., Poehlmann-Tynan J. (2021). Unmet mental health needs of jailed parents with young children. Fam. Relat..

[57] Berry P.E., Eigenberg H.M. (2003). Role strain and incarcerated mothers: Understanding the process of mothering. Women Crim. Justice.

[58] Arditti J., Few A. (2008). Maternal distress and women’s reentry into family and community life. Fam. Process.

[59] Skinner-Osei P., Stepteau-Watson D. (2018). A qualitative analysis of African American fathers’ struggle with reentry, recidivism, and reunification after participation in re-entry programs. J. Hum. Behav. Soc. Environ..

[60] Poehlmann J. (2005). Incarcerated mothers’ contact with children, perceived family relationships, and depressive symptoms. J. Fam. Psychol..

[61] Makariev D.W., Shaver P.R. (2010). Attachment, parental incarceration and possibilities for intervention: An overview. Attach. Hum. Dev..

[62] Bowlby J. (1982). Attachment and loss: Retrospect and prospect. Am. J. Orthopsychiatry.

[63] Colonnesi C., Draijer E.M., Jan J.M., Stams G., Van der Bruggen C.O., Bögels S.M., Noom M.J. (2011). The relation between insecure attachment and child anxiety: A meta-analytic review. J. Clin. Child. Adolesc. Psychol..

[64] Novero C.M., Booker Loper A., Warren J.I. (2011). Second-generation prisoners: Adjustment patterns for inmates with a history of parental incarceration. Crim. Justice Behav..

[65] Fowler J.C., Allen J.G., Oldham J.M., Frueh B.C. (2013). Exposure to interpersonal trauma, attachment insecurity, and depression severity. J. Affect. Disord..

[66] Borelli J.L., Goshin L., Joestl S., Clark J., Byrne M.W. (2010). Attachment organization in a sample of incarcerated mothers: Distribution of classifications and associations with substance abuse history, depressive symptoms, perceptions of parenting competency and social support. Attach. Hum. Dev..

[67] Miller S., Klockner K. (2019). Attachment styles and attachment based change in offenders in a prison Therapeutic Community. J. Forensic Psychol. Res. Pract..

[68] Eaton N.R., Keyes K.M., Krueger R.F., Balsis S., Skodol A.E., Markon K.E., Grant B.F., Hasin D.S. (2012). An invariant dimensional liability model of gender differences in mental disorder prevalence: Evidence from a national sample. J. Abnorm. Psychol..

[69] Drapalski A.L., Youman K., Stuewig J., Tangney J. (2009). Gender differences in jail inmates’ symptoms of mental illness, treatment history and treatment seeking. Crim. Behav. Ment. Health.

[70] Moloney K.P., van den Bergh B.J., Moller L.F. (2009). Women in prison: The central issues of gender characteristics and trauma history. Public Health.

[71] Arditti J.A., Smock S.A., Parkman T.S. (2005). It’s Been Hard to Be a Father: A qualitative exploration of incarcerated fatherhood. Father. A J. Theory Res. Pract. About Men Father..

[72] Clarke L., O’Brien M., Day R.D., Godwin H., Connolly J., Hemmings J., Van Leeson T. (2005). Fathering behind bars in English prisons: Imprisoned fathers’ identity and contact with their children. Father. J. Theory Res. Pract. Men Father..

[73] Lee C.B., Sansone F.A., Swanson C., Tatum K.M. (2012). Incarcerated fathers and parenting: Importance of the relationship with their children. Soc. Work Public Health.

[74] Day R.D., Acock A.C., Bahr S.J., Arditti J.A. (2005). Incarcerated fathers returning home to children and families: Introduction to the special issue and a primer on doing research with men in prison. Father. J. Theory Res. Pract. Men Father..

[75] Hairston C.F. (2002). Prisoners and Families: Parenting Issues during Incarceration. From Prison to Home: The Effects of Incarceration and Reentry on Children, Families and Communities Conference.

[76] Fowler C., Rossiter C., Dawson A., Jackson D., Power T. (2017). Becoming a “Better” father: Supporting the needs of incarcerated fathers. Prison J..

[77] Lanier C.S. (1993). Affective states of fathers in prison. Justice Q..

[78] Bartlett T.S., Flynn C.A., Trotter C.J. (2018). ‘They didn’t even let me say goodbye’: A study of imprisoned primary carer fathers’ care planning for children at the point of arrest in Victoria, Australia. Child. Care Pract..

[79] Tamburello A., Kaldany H., Dickert J. (2017). Correctional Mental Health Administration. Int. Rev. Psychiatry.

[80] Mongelli F., Georgakopoulos P., Pato M.T. (2020). Challenges and opportunities to meet the mental health needs of underserved and disenfranchised populations in the United States. Focus.

[81] Hoffmann H.C., Byrd A.L., Kightlinger A.M. (2010). Prison programs and services for incarcerated parents and their underage children: Results from a national survey of correctional facilities. Prison J..

[82] Newman C., Fowler C., Cashin A. (2011). The development of a parenting program for incarcerated mothers in Australia: A review of prison-based parenting programs. Contemp. Nurse.

[83] Purvis M. (2013). Paternal incarceration and parenting programs in prison: A review paper. Psychiatry Psychol. Law.

[84] Wilson K., Gonzalez P., Romero T., Henry K., Cerbana C. (2010). The effectiveness of parent education for incarcerated parents: An evaluation of parenting from prison. J. Correct. Educ..

[85] Butler M., Percy A., Hayes D., Devaney J. (2019). Designing prison-based parenting programs to maximize their outcomes. Int. J. Offender Ther. Comp. Criminol..

[86] Troy V., McPherson K.E., Emslie C., Gilchrist E. (2018). The feasibility, appropriateness, meaningfulness, and effectiveness of parenting and family support programs delivered in the criminal justice system: A systematic review. J. Child. Fam. Stud..

[87] Yasui M., Dishion T.J. (2007). The ethnic context of child and adolescent problem behavior: Implications for child and family interventions. Clin. Child. Fam. Psychol. Rev..

[88] Kumpfer K.L., Alvarado R., Smith P., Bellamy N. (2002). Cultural sensitivity and adaptation in family-based prevention interventions. Prev. Sci..

[89] Van Mourik K., Crone M.R., DeWolff M.S., Reis R. (2017). Parent training programs for ethnic minorities: A meta-analysis of adaptations and effect. Prev. Sci..

[90] Miller A.L., Weston L.E., Perryman J., Horwitz T., Franzen S., Cochran S. (2014). Parenting while incarcerated: Tailoring the strengthening families program for use with jailed mothers. Child. Youth Serv. Rev..

[91] LaRosa J.J., Rank M.G. (2003). Parenting education and incarcerated fathers. J. Fam. Soc. Work.

[92] Meek R. (2007). Parenting education for young fathers in prison. Child. Fam. Soc. Work.

[93] McCrudden E., Braiden H.J., Sloan D., McCormack P., Treacy A. (2014). Stealing the Smile from My Child’s Face: A Preliminary Evaluation of the “Being a Dad” Programme in a Northern Ireland Prison. Child. Care Pract..

[94] Fixsen D.L., Naoom S.F., Blase K.A., Friedman R.M., Wallace F., Burns B., Carter W., Paulson R., Schoenwald S., Barwick M. (2005). Implementation Research: A Synthesis of the Literature.

[95] McPherson K.E., Kerr S., Casey B., Marshall J. (2017). Barriers and facilitators to implementing functional family therapy in a community setting: Client and practitioner perspectives. J. Marital Fam. Ther..

[96] Morgan R.D., Steffan J., Shaw L.B., Wilson S. (2007). Needs for and barriers to correctional mental health services: Inmate perceptions. Psychiatr. Serv..

[97] Henson A. (2018). Strengthening evaluation research: A case study of an evaluability assessment conducted in a carceral setting. Int. J. Offender Ther. Comp. Criminol..

[98] Brown M., Bloom B. (2009). Reentry and renegotiating motherhood: Maternal identity and success on parole. Crime Delinq..

[99] Arditti J.A. (2018). Parental incarceration and family inequality in the United States. Prison. Punishm. Fam. New Sociol. Punishm..

[100] Muentner L., Charles P. (2020). A qualitative exploration of reentry service needs: The case of fathers returning from prison. Child. Fam. Soc. Work.

[101] Nhan J., Bowen K., Polzer K. (2017). The reentry labyrinth: The anatomy of a reentry services network. J. Offender Rehabil..

[102] Fehr L.M. (2004). Washington female offender re-entry programs combine transitional services with residential parenting. Correct. Today.

[103] Thalberg R.S. (2006). Family-based re-entry programming: A promising tool for reducing recidivism and mitigating the economic and societal costs of incarceration in California. SSRN.

[104] Listwan S.J., Cullen F.T., Latessa E.J. (2006). How to prevent prisoners re-entry programs from failing: Insights from evidence-based corrections. Fed. Probat..

[105] Enns P.K., Yi Y., Comfort M., Goldman A.W., Lee H., Muller C., Wakefield S., Wang E.A., Wildeman C. (2019). What percentage of Americans have ever had a family member incarcerated? Evidence from the family history of incarceration survey (FamHIS). Socius.

[106] Mitchell M.M., Spooner K., Jia D., Zhang Y. (2016). The effect of prison visitation on reentry success: A meta-analysis. J. Crim. Justice.

[107] Poehlmann J., Dallaire D., Loper A.B., Shear L.D. (2010). Children’s contact with their incarcerated parents: Research findings and recommendations. Am. Psychol..

[108] Buche J., Gaiser M., Rittman D., Beck A.J. (2018). Characteristics of the Behavioral Health Workforce in Correctional Facilities.

[109] Bagnall A.M., South J., Hulme C., Woodall J., Vinall-Collier K., Raine G., Kinsella K., Dixey R., Harris L., Wright N.M. (2015). A systematic review of the effectiveness and cost-effectiveness of peer education and peer support in prisons. BMC Public Health.

[110] Ray B., Watson D.P., Xu H., Salyers M.P., Victor G., Sightes E., Bailey K., Robison Taylor L., Bo N. (2021). Peer recovery services for persons returning from prison: Pilot randomized clinical trial investigation of SUPPORT. J. Subst. Abus. Treat..

[111] Gifford E.J., Kozecke L.E., Golonka M., Hill S.N., Costello E.J., Shanahan L., Copeland W.E. (2019). Association of parental incarceration with psychiatric and functional outcomes of young adults. JAMA Netw. Open.

[112] Tangney J.P., Stuewig J., Martinez A.G. (2014). Two faces of shame: The roles of shame and guilt in predicting recidivism. Psychol. Sci..

[113] Carlson J.R. (2009). Prison nurseries: A pathway to crime-free futures. Correct. Compend..

[114] Johnson S.M. (2019). Attachment Theory in Practice: Emotionally Focused Therapy (EFT) with Individuals, Couples, and Families.

[115] Swisher R.R., Waller M.R. (2008). Confining fatherhood: Incarceration and paternal involvement among nonresident White, African American, and Latino fathers. J. Fam. Issues.

[116] Binswanger I.A., Stern M.F., Deyo R.A., Heagerty P.J., Cheadle A., Elmore J.G., Koepsell T.D. (2007). Release from prison—A high risk of death for former inmates. New Engl. J. Med..

[117] Hong Chui W. (2016). Voices of the incarcerated father: Struggling to live up to fatherhood. Criminol. Crim. Justice Int. J..

[118] Dallaire D.H., Shlafer R.J., Goshin L.S., Hollihan A., Poehlmann-Tynan J., Eddy J.M., Adalist-Estrin A. (2021). COVID-19 and prison policies related to communication with family members. Psychol. Public Policy Law.

[119] Skora Horgan E., Poehlmann-Tynan J. (2020). In-home video chat for young children and their incarcerated parents. J. Child. Media.

[120] Larsen D., Hudnall Stamm B., Davis K., Magaletta P.R. (2004). Prison telemedicine and telehealth utilization in the United States: State and federal perceptions of benefits and barriers. Telemed. J. e-Health.

